# Assessing the relationship between ethical reasoning confidence and self-esteem among female nursing students for enhancing the quality of work life: A cross-sectional study

**DOI:** 10.1097/MD.0000000000037614

**Published:** 2024-04-05

**Authors:** Shaherah Yousef Andargeery, Sally Mohammed Farghaly Abdelaliem

**Affiliations:** a Nursing Management and Education Department, College of Nursing, Princess Nourah bint Abdulrahman University, Riyadh, Saudi Arabia.

**Keywords:** ethical confidence, ethical reasoning, nursing students, quality of life, self-esteem

## Abstract

To investigate the relationship between ethical reasoning confidence and self-esteem among female nursing students for enhancing the quality of work life. A necessary component of professional competence and a prerequisite for high-quality care is ethical reasoning confidence competence. As well as, self-esteem is the subjective perception of one’s own worth and significance. This was a descriptive correlational cross-sectional study design. Data was collected within a month starting from December 2022 to January 2023, and 164 nursing students were recruited from one College at a governmental university in Riyadh. Respondents completed the self-administered, online questionnaires. Measures included self-esteem, and ethical reasoning confidence questionnaires. Findings investigated via descriptive and inferential statistics as well as structured equation modeling to examine the mediating effect of self-esteem on behaviors and attitudes of the nursing students toward ethical reasoning confidence. Nursing students had a moderate perception regarding their self-esteem as well as their ethical reasoning confidence (Mean = 2.99, SD ± 0.58; and Mean = 3.57, SD ± 0.55, respectively). Data revealed that self-esteem was accounted for the prediction of 54% of positive variance of nursing students’ behaviors toward ethical reasoning confidence and 78% of the variance of their attitudes toward ethical reasoning confidence. Self-esteem is a significant determinant of nursing students’ behaviors and attitudes toward their ethical reasoning confidence. Further research is required to ascertain whether this approach enhances nursing students’ moral decision-making, moral reasoning, practical considerations, and acquaintance with ethical concerns.

## 1. Introduction

The foundation for tomorrow’s professional nurses will be today’s nursing students. They must be confident, forceful people with great self-esteem in order to assure competent and safe practice. With the development of technology, the growth of disease patterns, and the complexity of healthcare delivery, medical practices have been changing at a rapid rate in the healthcare system. Decision-making in various situations has gotten difficult as a result of changing role expectations for nurses.^[1]^ Given these difficulties, nurses must make morally sound decisions and accept responsibility for their actions while carrying out their tasks. If an ethical choice directly affects the health of patients and the standard of care given to them, nurses may feel stressed during the decision-making process.^[2]^ When nurses make moral judgments that diverge from their own moral standards, moral conflicts may result. Accordingly, ethical challenges have an impact on the level of burnout, the retention of nursing professionals, and the quality of nursing professional life.^[3]^

A necessary component of professional competence and a prerequisite for high-quality care is ethical reasoning confidence competence. According to Robbins (2011),^[4]^ ethical reasoning competence was described as “the courage to exhibit leadership in ethically ambiguous environments by confronting and engaging with ethical issues publicly and openly.” It was also discovered to be a subjective assessment of one’s own capacity to respond appropriately in specific circumstances.^[5]^ According to Olthuis et al (2007),^[6]^ self-esteem is the subjective perception of one’s own worth and significance. It can also be defined as the act of approving or disapproving a value depending on one’s own sense of value.^[7]^ In clinical practice, nurses consider their personal values and beliefs about what is right or proper. These values are therefore taken into account as the direction for carrying out the ethical behaviors when delivering safe practice, making the proper clinical decisions, and engaging with patients, colleagues, other healthcare professionals, and the general public.^[8,9]^ The nurse will be more motivated to consider their position and consequently more content with the nursing profession as they gain more confidence in their ability to make ethical decisions.^[10]^ In order to confidently make moral decisions, give patients the finest nursing care, and fight for their rights, nurses must possess strong moral sensibility and ethical knowledge.^[11]^

Due to a lack of ethical awareness and conflict resolution abilities, nursing students have reported difficulty handling moral problems and managing ethical conflicts, particularly during their clinical practices.^[5]^ According to Cameron et al (2016a),^[12]^ nursing students lack the necessary skills to strike a balance between the values at stake and understand the expected roles in any given circumstance. The rights of the patients and patient refusal of treatment are the most frequent ethical difficulties that nursing students are unaware of.^[13]^ Previous studies have shown that there are a variety of ethical problems among nursing students, such as an increase in conflicts between personal, professional, and organizational values related to insufficient resources, violations of patients’ rights and dignity, failure to uphold legal obligations, noncompliance with treatment standards and nursing standards, conflicts between policies and procedures with quality patient care, and unethical behavior of physicians or other nurses.^[12,14]^

Professional values vary from person to person and serve as the cornerstones of moral judgment and ethical behavior.^[15–17]^ According to the Theory of Self-Esteem and the Value Theory, a person’s decisions and actions are influenced by a variety of distinctive and important standards that define who they are as a person. These theories contend that internal conflict might arise when an individual’s internalized value system or the identity norm in a given context are inconsistent.^[18,19]^ Nurses should be aware of their own values, views, differences, and biases in order to manage stressful circumstances and disagreements efficiently.^[20]^ To ensure that ethical values are applied in nursing practice, professional values should be incorporated into nursing education through value-based behaviors.^[13]^

According to McWilliams and Nahavandi (2006),^[21]^ it is unclear how to effectively teach ethics so that nursing students gain ethical competence and assurance. The majority of ethical ideals and issues are taught either accidently or informally through clinical discussion and unplanned courses.^[22]^ According to the Pathways Commission’s recommendations,^[23]^ education should promote the ability to make moral decisions and use reasonable judgment. Loeb (2015)^[24]^ lists a number of methods for teaching ethics, including readings, case studies, role-playing, and self-reflection. Additionally, he believes that active learning techniques are superior to passive learning techniques.^[24]^ By employing more efficient methods to boost internalization and apply professional ideals to students’ performance, nurse educators play a crucial role.^[22]^

Future nurses should be prepared to make logical decisions based on ethical principles by teaching nursing students to be professionally educated, practically competent, and able to do so^[20,25,26]^ Nursing students begin to build their professional values when they enroll in universities, and this process continues when they work as clinical nurses. Developing decision-making skills and increasing ethical awareness at an early stage of education is crucial for better preparing nursing students to deal with ethical challenges during their clinical practice.^[27]^ There is a dearth of literature discussing the ethical reasoning confidence competency of nursing students, particularly in the middle east and its relation to their self-esteem. As a result, the objectives of this study are to: determine the perceived level of ethical reasoning confidence and self-esteem; and investigate the relationship between ethical reasoning confidence and self-esteem among nursing students in Saudi Arabia. So, the research question is; what is the relationship between ethical reasoning confidence and self-esteem among female nursing students for enhancing the quality of work life?

### 1.1. Research aim

To investigate the relationship between ethical reasoning confidence and self-esteem among female nursing students for enhancing the quality of work life.

## 2. Materials and method

### 2.1. Research design

This research was executed at a governmental University in Riyadh, Saudi Arabia, using a descriptive correlational cross-sectional study design.

#### 2.1.1. Setting.

The participants were recruited from the College of Nursing at one governmental university in Riyadh, Saudi Arabia. The College of Nursing is providing a nursing bachelor degree program which is an accredited nursing program by the Education and Training Evaluation Commission in Saudi Arabia and it provide other different nursing master programs including advanced nursing practitioner program. In addition, the College of nursing is one of the pioneering colleges to apply transition to practice program for nursing students to be enrolled in clinical practice while they are studying in 4th year and before the internship experience.

##### 2.1.1.1. Participants.

From a total population of 500 nursing students, the sample size for nursing students was estimated using Epi-info 7 based on a 5% variance, 95% confidence, and 0.80 power at a 0.5 significance level. The calculated sample size was 217 nursing students, but only 164 nursing students (75.6%) agreed to participate in the study by completing self-administered online questionnaire. Those students who volunteered to participate were from the College of Nursing at one governmental university in Riyadh, Saudi Arabia and met the following inclusion criteria: an adult of 18 years or older; and enrolled in the BSN program at the College of Nursing.

#### 2.1.2. Study tools.

##### 2.1.2.1. Socio-demographic and work-related questions.

The researchers developed a socio-demographic and work-related form that asked participants about their gender, age, study year, residence place and the most frequent problems that they were facing.

##### 2.1.2.2. Ethical Reasoning Confidence Scale (ERCS).

ERCS was used to assess nursing students’ perceptions toward ethical reasoning confidence. Smith et al, (2016)^[28]^ developed a scale that consists of 40 items divided into 4 parts of rank-order items and Likert-type scale which are as follows: The first part includes 10 items that are in a rank order for different skills from 1 = most important and 10 = least important to measure the perceived importance of ethical reasoning skills. The second part is a 5-point Likert-type scale that ranges from 1 = strongly disagree to 5 = strongly agree. The items are comprised of 2 factors that measure the importance and confidence in applying the ethical reasoning process. The third part of the scale includes 5 items that measure the frequency of applying, discussion, and engaging in ethical reasoning behaviors. This section is a 5-point Likert-type scale that ranged from 1 = Never to 5 = Daily. The more frequently used behaviors are indication of a more confident an individual is in ethical reasoning abilities. The fourth part contains 8 key questions that use a 5-point Likert-type scale and ranged from 1 = not at all important to 5 = very important to measure the importance of the 8 key questions in their ethical reasoning process. The questions help an individual to address complicated ethical situations. The total scale and the average score of the dimensions were computed, with higher scores indicating a higher level of ethical reasoning confidence.

##### 2.1.2.3. Self-esteem scale.

The self-esteem scale was used to evaluate nursing students’ level of self-esteem. It was created by Frank et al, (2008).^[29]^ The scale consists of 10 items. The scale is based on a 4-point Likert-type scale that ranged from 0 = strongly disagree to 3 = strongly agree. The items 3, 5, 8, 9, 10 are reversed coded. The total score ranges from 0 to 30. Scores between 15 and 25 are considered normal self-esteem, while scores higher than 25 indicate a higher level of self-esteem and scores below 15 reflect low self-esteem.

### 2.2. Validity and reliability

All instruments have been verified as reliable using Cronbach’s alpha coefficient to estimate item internal consistency. The scales confirmed reliability with values of 0.870 and 0.849 for ERCS and Self-Esteem Scale, respectively. In addition, a pilot study with 10% of nurses was undertaken to support the tools’ validity and reliability.

### 2.3. Data collection

The College administration granted written permission for data collection. For the study, nursing students were given link with the online survey. To assess the feasibility of the study, 10% of the study sample (n = 16) agreed to participate in a pilot study; participants in the pilot study were excluded from the study sample. The pilot study showed that the study tools were clear and that no changes were necessary. The questionnaires took between 10-15 minutes to complete. From December 2022 to January 2023, data was gathered over a month period.

### 2.4. Ethical considerations

The IRB Approval (IRB; 22-0491) was taken from the educational government institution’s Review Board and the directors of the study settings provided official permission to collect the necessary data. The data’s confidentiality was maintained. Prior to data collection, the informed consents were given by the nursing students’ written agreement to participate in the study. The participants were given the option to leave the study at any time.

### 2.5. Data analysis

Frequencies and percentages were used to present socio-demographic characteristics; mean and standard deviation (SD) were used to show continuous variables. To investigate the type of relationship perceived by nursing students between ethical reasoning confidence and self-esteem, Pearson correlation coefficient analysis (*r*) was used. Inferential statistics were used to analyze the study’s findings (Pearson correlation coefficient and Regression analysis [*R*^2^]). All statistical analyses were carried out with an alpha error of 0.05. All statistical analyses were performed using IBM SPSS Statistics version 23. All *P* values reported are two-tailed, with statistical significance determined at the .05 level. The mediating effect of self-esteem was examined using a structured equation modeling. The researchers considered the self-esteem as a mediator variable, behaviors, reasoning and attitudes toward ethical reasoning confidence as an dependent variables. All statistical analyses were conducted using IBM SPSS Statistics version 23 and IBM SPSS AMOS version 23 (Armonk, NY). All reported *P* values are 2-tailed, and .05 level was used for statistical significance.

## 3. Results

Descriptive statistics were implemented for continuous variables using the mean and standard deviation, while the categorical variables were implemented using frequencies and percentages (Table 1). The total number of participants in this study is 164 students. Participants of this study were all female. The mean age of the participants is (Mean = 19.80, SD = 1.47), of them 78 participants (47.6%) were under the age of 20, 80 participants (48.8%) were between the age of 20 and 22, and only 6 participants (3.7%) were 23 years old or over. All the participants are students in the College of Nursing, of them 60 participants (36.6%) were studying in first year, 33 participants (20.1%) were studying in the second year, 40 participants (24.4%) were studying in the third year, 18 participants (11%) were studying in the fourth year, and 13 participants (7.9%) were intern students. Second, third, fourth year, and intern nursing students have enrolled with clinical training throughout their education as a mandatory requirement in their educational curriculum. The majority of the participants (162 students, 98.8%) indicated that they receive training in clinical areas, while only 2 participants (1.2%) indicated that they did not have previous training. Most of the participants (149 students, 90%) live with their family, while only 15 participants (9.1%) indicated that they live in dormitory.

**Table 1:** Socio-demographic characteristics of participants (n = 164).

Socio-demographics characteristics	N	%
Age		
<20	78	47.6
20–22	80	48.8
≥ 23	6	3.7
Mean ± SD	19.80 ± 1.47	
Gender		
Male	0	0
Female	164	100
Study year		
1st	60	36.6
2nd	33	20.1
3rd	40	24.4
4th	18	11.0
Intern	13	7.9
Current clinical training enrollment		
Has took training	162	98.8
Didn’t trainee	2	1.2
Residence place		
Dormitory	15	9.1
With family	149	90.9

The distribution of study participants to the problems are presented in Table 2. Most of the participants (159 students, 97%) disclosed that they have an interest in the field of nursing. Around one third of the participants (39 students, 23.8%) revealed that they have financial difficulty. A quarter of the participants (27 students, 16.5%) indicated that they have physical health problems. Only 4.3% of the participants revealed that they complained of any mental problem. Around a quarter of the participants (21 students, 12.8%) disclosed that they have psychiatric disorder. Ten participants (6.1%) indicated that they have chronic illness.

**Table 2:** Distribution of the studied sample according to percentage of problems occurrence (n = 164).

Problem occurrence	No		Yes	
	N	%	N	%
Do you have an interest in the field of nursing?	5	3.0	159	97.0
Do you have any financial difficulty?	125	76.2	39	23.8
Do you have any physical health problem?	137	83.5	27	16.5
Do you complain of any mental problem?	157	95.7	7	4.3
Do you have any psychiatric disorder?	143	87.2	21	12.8
Do you have any chronic illness?	154	93.9	10	6.1

Table 3 shows the means and standard deviation for the Rosenberg’s Self-Esteem Scale. The overall Rosenberg’s Self-Esteem Scale showed that the participants reported a moderate level of self-esteem (Mean = 2.99, SD ± 0.58). The highest 3 items contributed to the participants’ self-esteem are feeling that they have a number of good qualities (Mean = 3.43, SD ± 0.63), they are able to do things as well as most other people (Mean = 3.25, SD ± 0.75), and taking a positive attitude toward themselves (Mean = 3.21, SD ± 0.76). The lowest 3 items of self-esteem that were identified by the participants where they wish they could have more respect for themselves (Mean = 2.18, SD ± 1.02), they think they are no good at all (Mean = 2.79, SD ± 0.98), and they feel useless at times (Mean = 2.82, SD ± 0.99).

**Table 3:** Mean Score and perception level of participants’ self-esteem (n = 164).

Self-esteem	Mean ± SD
1. I feel that I am a person of worth, at least on an equal plane with others.	3.14 ± 0.81
2. I feel that I have a number of good qualities	3.43 ± 0.63
3. All in all, I am inclined to feel that I am a failure.	3.07 ± 0.88
4. I am able to do things as well as most other people.	3.25 ± 0.75
5. I feel I do not have much to be proud of.	2.89 ± 1.04
6. I take a positive attitude toward myself.	3.21 ± 0.76
7. On the whole, I am satisfied with myself.	3.16 ± 0.89
8. I wish I could have more respect for myself.	2.18 ± 1.02
9. I certainly feel useless at times.	2.82 ± 0.99
10. At times I think I am no good at all.	2.79 ± 0.98
Overall self-esteem	2.99 ± 0.58

SD = standard deviation.

Table 4 represents the means and standard deviations for the rank-order ethical reasoning skills. The participants ranked artistic skills as the first most important skills amongst the other 9 skills (Mean = 4.62, SD ± 3.50). The participants ranked budgeting skills as the second most important skills after artistic skills (Mean = 4.93, SD ± 3.0). Critical thinking skills were ranked as the third most important skills by the participants of this study (Mean = 5.10, SD ± 2.47). One of the main variables of this study is ethical reasoning skills which were ranked as the fourth important skills (Mean = 5.21, SD ± 2.37). The 3 least important skills that were ranked by the participants are oral communication skills (Mean = 5.77, SD ± 2.29), time management skills (Mean = 5.93, SD ± 3.0), and writing skills (Mean = 6.55, SD ± 3.29).

**Table 4:** Mean score and level of participants’ ethical reasoning related skills (n = 164).

Ethical reasoning	Mean ± SD	Ranking most important
Rank-order the following skills from 1 (Most important) to 10 (Least important)		
1. Artistic Skills	4.62 ± 3.50	1
2. Budgeting Skills	4.93 ± 3.0	2
3. Critical Thinking Skills	5.10 ± 2.47	3
4. Ethical Reasoning Skills	5.21 ± 2.37	4
5. Interpersonal Skills	5.41 ± 2.42	5
6. Oral Communication Skills	5.77 ± 2.29	8
7. Organization Skills	5.73 ± 2.59	6
8. Programming Skills	5.75 ± 3.10	7
9. Time-management Skills	5.93 ± 3.0	9
10. Writing Skills	6.55 ± 3.29	10

SD = standard deviation.

The mean and standard deviations for Attitudes toward ethical reasoning skills, ethical reasoning behaviors, ethical reasoning process, and the overall ethical reasoning confidence are presented in Table 5. The participants of this study experienced a moderate level of ethical reasoning confidence (Mean = 3.57, SD ± 0.55).

**Table 5:** Mean Score and level of participants’ ethical reasoning (n = 164).

Ethical reasoning	Mean ± SD
Attitudes toward ethical reasoning skills	
1. Ethical reasoning skills are important to me	4.24 ± 0.87
2. Having good ethical reasoning skills will be useful in my future jobs	4.54 ± 0.71
3. Every university should teach ethical reasoning	4.14 ± 0.91
4. I believe ethical reasoning is a valuable skillset	4.29 ± 0.73
5. Ethical reasoning skills are beneficial to making difficult life choices	4.24 ± 0.83
6. I am not a PNU student	1.40 ± 0.97
7. When faced with an ethical dilemma, I feel confident in making an appropriate decision	3.61 ± 1.07
8. I feel prepared to deal with complex life situations that involve ethics	3.66 ± 1.02
9. I am comfortable applying my ethical reasoning skills to real life situations	4.00 ± 0.91
10. I can actively participate in a discussion about ethics	3.81 ± 1.08
11. I am capable of evaluating my options using an ethical reasoning process	4.02 ± 0.85
12. I can state from memory the 8 key questions of ethical reasoning	3.10 ± 1.24
13. When faced with an ethical situation, I can correctly identify the most relevant key questions	3.52 ± 1.10
14. I can weigh and balance the relevant key questions to make an informed decision	3.61 ± 1.08
15. I can apply the 8-key-question ethical reasoning framework to aspects of my personal life	3.57 ± 1.05
16. I can apply the 8-key-question ethical reasoning framework to aspects of my professional Life	3.54 ± 1.05
17. I can apply the 8-key-question ethical reasoning framework to aspects of my civic life	3.52 ± 1.05
Overall Attitudes toward ethical reasoning skills	3.69 ± 0.64
Behaviors	
18. How often do you think about ethical issues?	2.59 ± 1.20
19. How often do you apply ethical reasoning to make a decision?	3.10 ± 1.24
20. How often do you think about ethics when grappling with complex situations?	3.62 ± 1.24
21. How often do you engage in ethical reasoning when giving advice to others?	3.29 ± 1.27
22. How often do you discuss real-life ethical dilemmas with others?	2.83 ± 1.22
Overall behaviors	3.09 ± 0.94
From your current perspective, please indicate how important each of the key questions is in your ethical reasoning process	
23. Empathy	3.19 ± 1.69
24. Fairness	4.34 ± 1.37
25. Character	3.15 ± 1.77
26. Liberty	3.23 ± 1.74
27. Rights	4.55 ± 1.21
28. Responsibilities	4.12 ± 1.59
29. Outcomes	3.68 ± 1.70
30. Authority	2.55 ± 1.39
Overall reasoning process	3.60 ± 0.93
Overall ethical reasoning	3.57 ± 0.55

SD = standard deviation.

Participants perceived a moderate level of attitudes toward ethical reasoning skills (Mean = 3.69, SD ± 0.64). The highest attitude toward ethical reasoning skills revealed by the participants is having good ethical reasoning skills will be useful in their future jobs (Mean = 4.54, SD ± 0.71). The other 3 top attitudes toward ethical reasoning skills perceived by the participants are they believe that ethical reasoning is a valuable skillset (Mean = 4.29, SD ± 0.73), are beneficial to making difficult life choices (Mean = 4.24, SD ± 0.83), and are important to them (Mean = 4.24, SD ± 0.87). One of the lowest attitudes toward ethical reasoning skills perceived by the participants is that they can state from memory the 8 key questions of ethical reasoning (Mean = 3.10, SD ± 1.24). The other attitudes that were contributed to decease the ethical reasoning skills was when the participants faced with an ethical situation, they can correctly identify the most relevant key questions and they can apply the 8-key-question ethical reasoning framework to aspects of their civic life (Mean = 3.52, SD ± 1.10).

The overall behaviors mean score indicated that participants reported a moderate level of ethical reasoning behaviors (Mean = 3.09, SD ± 0.94). The highest ethical reasoning behaviors was how often do the participants think about ethics when grappling with complex situations (Mean = 3.62, SD ± 1.24). The least ethical reasoning behaviors was how often do they think about ethical issues (Mean = 2.59, SD ± 1.20). The participants reported a moderate level of reasoning process (Mean = 3.60, SD ± 0.93). They perceived that the rights as the most important item in their ethical reasoning process (Mean = 4.55, SD ± 1.21), while they indicated the authority as the least important item in their ethical reasoning process (Mean = 2.55, SD ± 1.39).

Pearson correlation coefficient was implemented to examine the relationship between the study variables (Table S1, Supplemental Digital Content, http://links.lww.com/MD/L992). There was a statistically significant correlation between attitudes toward ethical reasoning and self-esteem (*r* = 0.790, *P* < .001), meaning that there is a strong positive correlation between both variables. In addition, there was a statistically significant strong positive correlation between ethical reasoning behaviors and self-esteem (*r* = 0.562, *P* < .001). There is a statistically significant moderate correlation between ethical reasoning process and self-esteem (*r* = 0.469, *P* < .001). There is a statistically significant strong positive correlation between the overall ethical reasoning confidence and self-esteem. A statistically significant moderate correlation was found between attitudes toward ethical reasoning and ethical reasoning behaviors (*r* = 0.447, *P* < .001). However, there was a statically significant weak correlation between attitudes toward ethical reasoning and ethical reasoning process (*r* = 0.171, *P* = .029). The overall ethical reasoning confidence has a statistically significant strong correlation with the attitudes toward ethical reasoning (*r* = 0.861, *P* < .001). A non-statistically significant correlation was found between ethical reasoning process and ethical reasoning behaviors (*r* = 0.068, *P* = .388). The overall ethical reasoning confidence has a statistically significant strong correlation with ethical reasoning behaviors (*r* = 0.609, *P* < .001). ethical reasoning process has a statistically significant strong correlation with the overall ethical reasoning confidence.

A stepwise hierarchical linear regression analysis was conducted to examine if the attitudes toward ethical reasoning, ethical reasoning behaviors, and ethical reasoning process predict self-esteem as perceived by the nursing students (Table S2, Supplemental Digital Content, http://links.lww.com/MD/L993). The model showed that the variables are statistically significant predictors of self-esteem *F* = 206.730, *P* < .001, and accounted for 79% of the variance in self-esteem (*r*^2^ = 0.790). The strongest predictor of model set was attitudes toward ethical reasoning (β = 0.613, *P* < .001), followed by ethical reasoning process (β = 0.346, *P* < .001), then ethical reasoning behaviors (β = 0.264, *P* < .001). Generally, all the independent variables had a direct significant influence on self-esteem. Thus, nursing students who perceived higher attitudes toward ethical reasoning, ethical reasoning process, and ethical reasoning behaviors reported greater self-esteem. Among the independent variables, attitudes toward ethical reasoning had greater influence on self-esteem.

The results of the structure equation modeling, Figure S1, Supplemental Digital Content, http://links.lww.com/MD/L994 showed the path analysis model drawn with SPSS-AMOS clarifying the standardized regression weights of the structured equation modeling (Model χ^2^ = 2.610; *P* < .001); Model fit parameters (CFI = 0.646; IFI = 0.651; RMSEA = 0.100). Self-esteem accounted for the prediction of 54% of positive variance of nursing students’ behaviors toward ethical reasoning confidence and 78% of the variance of their attitudes toward ethical reasoning confidence.

## 4. Discussion

It is crucial to look into how self-esteem and ethical reasoning confidence are related among female nursing students in order to improve their quality of life and ethical reasoning and decision-making. and nursing abilities. Undergraduates studying nursing regularly encounter novel, unusual, and difficult circumstances. They move into a new setting, thus they must swiftly adapt in order to carry out their academic duties and adjust to the sociocultural shifts. Being away from home for the first time, alterations to daily routines, increased responsibility and organization required, exposure to professional practice, demands, expectations, and a lack of support for leaving friends and family are a few problems. The results of the current study showed that nursing students’ had a moderate perceptions regarding their self-esteem. This is in line with earlier studies^[30,31]^ that discovered nursing staff perceived their self-esteem and self-efficacy at levels ranging from moderate to high. They may be able to perform tasks on par with the majority of people, possess a variety of positive traits and skills, have a high level of artistic skill perception, have a positive outlook on life, and believe they are at least on par with other people in terms of value. The results of the present study are in agreement with those of Shrestha (2019)^[32]^ and Nandan (2021),^[33]^ who found that the majority of nursing students exhibit moderate assertiveness and self-esteem.

The current study’s findings regarding ethical reasoning confidence showed that nursing students’ had a moderate perception of their self-esteem. This may be related to their high belief in ethical reasoning as a valuable skillset, the significance of ethical reasoning skills in helping nursing students make difficult life decisions, the fact that they learned about ethical dilemmas and ethical decision making within the curriculum of nursing, and the fact that they were capable of evaluating the situation from an ethical standpoint. This result was in line with earlier research,^[34,35]^ which demonstrated that debate-based ethics instruction for undergraduate nursing students is highly successful in fostering moral judgment and the capacity for making ethical decisions. The use of the problem-based learning approach in ethics education promotes the moral development of nursing students, according to the current result, which can be credited to the nurse educators. In order to improve moral sensitivity, Baykara et al (2015) discovered that it was crucial to identify the environmental and personal components that may have an impact on moral sensitivity. They also found that freshmen and sophomore nursing students should receive nursing ethics education. However, Choe et al (2013)^[36]^ found that junior and senior students are typically taught nursing ethics in undergraduate nursing curriculum. Therefore, it is vital to reform nursing curricula so that first-year and second-year nursing students receive ethics instruction. In addition, more research is required to determine the best timing and length of education for improving moral sensitivity,^[37]^ as well as pedagogical techniques to accomplish so. When confronted with a moral conundrum, students typically struggle to reach idealistic moral conclusions if their moral reasoning system has been compromised. However, by focusing on the core of issues and acquiring knowledge that supports decision-making while engaging in interactive and cooperative communication, students can maintain their idealistic judgment while developing reflective reasoning skills to resolve cognitive conflicts through argumentation.^[38]^ Making moral decisions in difficult situations heavily relies on idealistic moral judgment, which involves properly choosing the best and most practical course of action as a professional. As a result, clinical nurses should get ongoing education to improve their capacity for idealistic moral judgment.

the claim that among nursing students, there was a statistically significant association between ethical judgment and self-esteem. Additionally, self-esteem was responsible for the prediction of a favorable variance in the actions and attitude of nursing students toward ethical reasoning. This result showed that nursing students’ readiness, abilities, and different skills, such as artistic, critical thinking, reasoning, and decision-making skills, for facing and managing various situations are affected when they believe in their person of worth with a sense of equality, have various positive qualities, disagree with the failure condition, and have a sense of ability to do things effectively. Iacobucci et al (2013)^[5]^ conducted a study with a convenience sample of 47 senior nursing students from the United States and asked them to complete surveys regarding their perceived level of confidence in making ethical decisions, their level of internalized professional nursing values, and their level of self-esteem using the Rosenberg’s Self-Esteem Scale. The levels of self-esteem and professional nursing values of nursing students were shown to be significantly positively correlated (*P* < .05). Similar findings were made by Fagerberg (2004)^[39]^ who discovered that nursing students strongly appreciated patient advocacy and nursing practice that was governed by the principles of fidelity, privacy, secrecy, and respect. Additionally, participants felt it was highly important to address healthcare professionals who were engaging in dubious or unethical behavior and reported having somewhat high levels of perceived trust in their ethical decision-making. These results corroborate past research that shows self-esteem encourages nurses to act as patient advocates when faced with value conflict and helps them by reconciling organizational and personal values within the context of professional identity. Furthermore, Yildirim et al (2017)^[40]^ reported that social support and self-esteem have an impact on the ethical decision-making abilities of nursing students.

## 5. Conclusion

In light of the present study findings, it was concluded that nursing students had a moderate perception regarding their self-esteem, as well as, they had a moderate behavior and attitude toward their ethical reasoning confidence. There was a statistically significant positive correlation between ethical reasoning confidence and their self-esteem, as perceived by nursing students. In addition, self-esteem had a strong effect on the variance of the nursing students’ behavior and attitude regarding their ethical reasoning process and confidence. Nurse educators should take initiation to improve high assertiveness and self-esteem of nursing students.

## 6. Implications of the study

The present study’s significant practice implications should be taken into account. The results show that nursing students’ mental health has to be improved, favoring their sense of worth and confidence in their abilities. Since these constructs have been shown to be connected and play a crucial role in both the individual’s personal life and the process of becoming a professional, the educational authorities need to be aware of this vulnerability as well as the related elements. Promoting them in a university environment is crucial to aid in the development of young people who are healthy and productive. The results also indicate a broader look at future intervention research studies that concentrate on the students’ self-efficacy and self-esteem in order to help them handle challenges in a healthier and more effective way, preventing unfavorable consequences and promoting learning. This study was constrained by its cross-sectional design and inability to evaluate the undergraduate students during the duration of the program in order to pinpoint the most crucial moments. It is important to emphasize how precarious the decision to use a generic tool to gauge the participants’ self-efficacy was. Additionally, because the instruments used in this study are subjective and self-reported, respondents must reflect on their responses and gain a better understanding of themselves. In addition, even with the guarantee of anonymity, the participant could feel awkward stating negative thoughts about themselves and a decreased capacity to do tasks satisfactorily. Further research is required to ascertain whether this approach enhances nursing students’ moral decision-making, moral reasoning, practical considerations, and acquaintance with ethical concerns.

## Acknowledgments

The authors extend their appreciation to the Deputyship for Research and Innovation, Ministry of Education in Saudi Arabia for funding this research work through the project number RI-44-0020.

## Author contributions

**Conceptualization:** Sally Mohammed Farghaly Abdelaliem.

**Data curation:** Sally Mohammed Farghaly Abdelaliem.

**Formal analysis:** Sally Mohammed Farghaly Abdelaliem.

**Funding acquisition:** Shaherah Yousef Andargeery.

**Investigation:** Sally Mohammed Farghaly Abdelaliem.

**Methodology:** Sally Mohammed Farghaly Abdelaliem.

**Project administration:** Sally Mohammed Farghaly Abdelaliem.

**Resources:** Shaherah Yousef Andargeery.

**Software:** Sally Mohammed Farghaly Abdelaliem.

**Supervision:** Sally Mohammed Farghaly Abdelaliem.

**Writing – original draft:** Sally Mohammed Farghaly Abdelaliem.

**Writing – review & editing:** Sally Mohammed Farghaly Abdelaliem.

## Supplementary Material







## References

[1] Yoo M-S 손기철. Effects of nursing ethics education on biomedical ethics awareness, moral sensitivity and moral judgment of nursing students. J Korea Bioethics Assoc. 2011;12:1–10.

[2] Han S-S Ahn S Kim J. Validation of a Korean version of the moral sensitivity questionnaire. Nurs Ethics. 2010;17:99–105. 20089629 10.1177/0969733009349993

[3] Poorchangizi B Borhani F Abbaszadeh A. Professional values of nurses and nursing students: a comparative study. BMC Med Educ. 2019;19:438. 31775723 10.1186/s12909-019-1878-2 PMC6882014

[4] Robbins J. Defining integrity for individuals and organizations: a cognitive-linguistic modeling approach. In: Handbook of Research on Teaching Ethics in Business and Management Education. IGI Global Publishing Tomorrow's Research Today: United States; 2011.

[5] Iacobucci TA Daly BJ Lindell D. Professional values, self-esteem, and ethical confidence of baccalaureate nursing students. Nurs Ethics. 2013;20:479–90. 23166146 10.1177/0969733012458608

[6] Olthuis G Lehet C Dekkers W. Why hospice nurses need high self-esteem. Nurs Ethics. 2007. Accessed on November, 2023. Available at: http://sdl.edu.sa/middleware/Default.aspx?USESDL=true&PublisherID=AllPublishers&BookURL=https://sdl.idm.oclc.org/login?url=https://search.ebscohost.com/login.aspx?direct=true&db=edsoai&AN=edsoai.on1284166394&site=eds-live. 10.1177/0969733007071359 17334171

[7] Rosenberg M. Society and the Adolescent Self-Image. Princeton University Press: New Jersey; 1965. Accessed on November, 2023. Available at: http://www.jstor.org/stable/j.ctt183pjjh.

[8] Blais K Hayes J. Professional Nursing Practice: Concepts and Perspectives. University of Northern Colorado; 2015. Available at: https://www.pearson.com/en-us/subject-catalog/p/professional-nursing-practice-concepts-and-perspectives/P200000000799/9780137518531.

[9] Kaya H Işik B Şenyuva E. Personal and professional values held by baccalaureate nursing students. Nurs Ethics. 2017;24:716–31. 26822298 10.1177/0969733015624488

[10] Lim M-H. Effects of moral sensitivity and critical thinking disposition on perceived ethical confidence in nursing students. J Korea Acad Ind Coop Soc. 2016;17:610–8. Accessed on November, 2023. Available at: http://sdl.edu.sa/middleware/Default.aspx?USESDL=true&PublisherID=AllPublishers&BookURL=https://sdl.idm.oclc.org/login?url=https://search.ebscohost.com/login.aspx?direct=true&db=edsair&AN=edsair.doi...........3f25285c191987fc394e4977d6632c0a&site=eds-live.

[11] Lim M. Converged study of influencing factors on perceived ethical confidence in nurses. J Korean Chem Soc. 2017;8:75–84.

[12] Cameron ME Schaffer M Park H-A. Nursing students’ experience of ethical problems and use of ethical decision-making models. SAGE Publications. 2016;8:432–47. 10.1177/096973300100800507 16004097

[13] Parandeh A Khaghanizade M Mohammadi E. Factors influencing development of professional values among nursing students and instructors: a systematic review. Glob J Health Sci. 2015;7:1–12. 10.5539/gjhs.v7n2p284 PMC4796667 25716397

[14] Aroskar MA Moldow DG Good CM. Nurses’ voices: policy, practice and ethics. Nurs Ethics. 2004;11:266–76. 15176640 10.1191/0969733004ne694oa

[15] Eddy DM Elfrink V Weis D. Importance of professional nursing values: a national study of baccalaureate programs. J Nurs Educ. 1994;33:1–9. 10.3928/0148-4834-19940601-07 8046517

[16] LeDuc K Kotzer AM. Bridging the gap: a comparison of the professional nursing values of students, new graduates, and seasoned professionals. Nurs Educ Perspect. 2009;30:279–84. 19824236

[17] Weis D Schank MJ. Toward building an international consensus in professional values. Nurse Educ Today. 1997;17:366–9. 9370627 10.1016/s0260-6917(97)80096-2

[18] Cast AD Burke PJ. A theory of self-esteem. Soc Forces. 2002;80:1041–68.

[19] Rokeach M. The nature of human values. In: The Nature of Human Values. Free Press, Oxford Academics: United States; 1973.

[20] Kim K Han Y Kim JS. Korean nurses’ ethical dilemmas, professional values and professional quality of life. Nurs Ethics. 2015;22:1–7. 10.1177/0969733014538892 24964868

[21] McWilliams V Nahavandi A. Using live cases to teach ethics. J Bus Ethics. 2006;67:421–33.

[22] Bijani M Tehranineshat B Torabizadeh C. Nurses’, nursing students’, and nursing instructors’ perceptions of professional values: a comparative study. Nurs Ethics. 2019;26:870–83. 28905676 10.1177/0969733017727153

[23] Behn BK Ezzell WF Murphy LA. The pathways commission on accounting higher education: charting a national strategy for the next generation of accountants. Issues Account Educ. 2012;27:595–600.

[24] Loeb SE. Active learning: an advantageous yet challenging approach to accounting ethics instruction. J Bus Ethics. 2015;127:221–30.

[25] Kim EA Kim NY. Mediation effect of biomedical ethics awareness between moral sensitivity and perceived ethical confidence among nursing students. J Korean Acad Nurs Adm. 2020;26:511–20.

[26] Kim MS. Influence of moral sensitivity and ethical values on biomedical ethics awareness of nursing students. J Korean Acad Soc Nurs Educ. 2015;21:382–92.

[27] Lim M-H. Effects of moral sensitivity and critical thinking disposition on perceived ethical confidence in nursing students. J Korea Acad Ind Coop Soc. 2016;17:610–8.

[28] Franck E De Raedt R Barbez C. Psychometric properties of the Dutch Rosenberg self-esteem scale. Psychol Belg. 2008;48:25–35.

[29] Smith KL Bashkov BM Fulcher KH. Assessing Attitudes Toward Ethical Reasoning: Examining the Factor Structure of the Survey of Ethical Reasoning. Harrisonburg, VA: James Madison University; 2014.

[30] Ribeiro RM Bragiola JVB Eid LP. Impact of self-esteem and of the sociodemographic factors on the self-efficacy of undergraduate nursing students. Texto Contexto-Enfermagem. 2019;29:29.

[31] Chung JM Robins RW Trzesniewski KH. Continuity and change in self-esteem during emerging adulthood. J Pers Soc Psychol. 2014;106:469–83. 24377355 10.1037/a0035135 PMC4049296

[32] Shrestha S. Assertiveness and self-esteem among nursing students of Manipal College of Medical Science of Pokhara, Nepal. J Chitwan Med Coll. 2019;9:54–9.

[33] Nandan L. Self-Esteem among the Nursing students of selected Institutions of Noida. Int J Nurs Educ Res. 2021;9:91–5.

[34] Greco S Lewis EJ Sanford J. Ethical reasoning debriefing in disaster simulations. J Prof Nurs. 2019;35:124–32. 30902404 10.1016/j.profnurs.2018.09.004

[35] Kim WJ Park JH. The effects of debate-based ethics education on the moral sensitivity and judgment of nursing students: a quasi-experimental study. Nurse Educ Today. 2019;83:104200. 31491613 10.1016/j.nedt.2019.08.018

[36] Choe K Kang Y Lee WY. Bioethics education of nursing curriculum in Korea: a national study. Nurs Ethics. 2013;20:401–12. 23295639 10.1177/0969733012466003

[37] Hoseini M Evadi M Farsi Z. The effect of moral motivation training on moral sensitivity in the nurses of military hospitals. Mil Caring Sci. 2018;4:249–57.

[38] Yeom HA Ahn SH Kim SJ. Effects of ethics education on moral sensitivity of nursing students. Nurs Ethics. 2016;24:644–52. 26811393 10.1177/0969733015622060

[39] Fagerberg I. Registered nurses’ work experiences: personal accounts integrated with professional identity. J Adv Nurs. 2004;46:284–91. 15066109 10.1111/j.1365-2648.2004.02988.x

[40] Yildirim N Karaca A Cangur S. The relationship between educational stress, stress coping, self-esteem, social support, and health status among nursing students in Turkey: a structural equation modeling approach. Nurse Educ Today. 2017;48:33–9. 27701030 10.1016/j.nedt.2016.09.014

