# Supplementary material for: Assessing the relationship between ethical reasoning confidence and self-esteem among female nursing students for enhancing the quality of work life: A cross-sectional study
Source: Medicine (Baltimore). 2024 Apr 5;103(14):e37614. doi: 10.1097/MD.0000000000037614 (PMC10994480; doi:10.1097/MD.0000000000037614)
Supplement: Supplementary file 1 [file medi-103-e37614-s001.docx]

Supplementary Table 1**: Correlation between self- esteem and ethical reasoning (n=164)**

| **Variables** |  | **Self-Esteem** | **Attitudes** | **Behaviors** | **Reasoning** |
| --- | --- | --- | --- | --- | --- |
| **Self-Esteem** | **r** |  |  |  |  |
|  | **p** |  |  |  |  |
| **Attitudes** | **r** | 0.790^*^ |  |  |  |
|  | **p** | <0.001^*^ |  |  |  |
| **Behaviors** | **r** | 0.562^*^ | 0.447^*^ |  |  |
|  | **p** | <0.001^*^ | <0.001^*^ |  |  |
| **Reasoning** | **r** | 0.469^*^ | 0.171^*^ | 0.068 |  |
|  | **p** | <0.001^*^ | 0.029^*^ | 0.388 |  |
| **Overall Ethical Reasoning** | **r** | 0.890^*^ | 0.861^*^ | 0.609^*^ | 0.581^*^ |
|  | **p** | <0.001^*^ | <0.001^*^ | <0.001^*^ | <0.001^*^ |

**r: Pearson coefficient**

*: Statistically significant at p ≤ 0.05
