# Supplementary material for: Assessing the relationship between ethical reasoning confidence and self-esteem among female nursing students for enhancing the quality of work life: A cross-sectional study
Source: Medicine (Baltimore). 2024 Apr 5;103(14):e37614. doi: 10.1097/MD.0000000000037614 (PMC10994480; doi:10.1097/MD.0000000000037614)
Supplement: Supplementary file 2 [file medi-103-e37614-s002.docx]

Supplementary Table 2**: Hierarchical Linear Regression Analysis (Stepwise) Showing the Effect of Ethical Reasoning on Self-Esteem (n=164)**

| Variable | B | Beta | t | p | 95% CI | |
| --- | --- | --- | --- | --- | --- | --- |
|  |  |  |  |  | **LL** | **UL** |
| Attitudes | 0.551 | 0.613 | 15.113^*^ | <0.001^*^ | .479 | 0.623 |
| Reasoning | 0.214 | 0.346 | 9.525^*^ | <0.001^*^ | .170 | 0.259 |
| Behaviors | 0.161 | 0.264 | 6.602^*^ | <0.001^*^ | .113 | 0.209 |
| R^2^=0.790,F= 206.730^*^,p<0.001^*^ | | | | | | |

F,p: f and p values for the model

R^2^: Coefficient of determination

B: Unstandardized Coefficients

Beta: Standardized Coefficients

t: t-test of significance

CI: Confidence Interval

LL: Lower limit UL: Upper Limit

*: Statistically significant at p ≤ 0.05
