# Supplementary material for: Assessing the relationship between ethical reasoning confidence and self-esteem among female nursing students for enhancing the quality of work life: A cross-sectional study
Source: Medicine (Baltimore). 2024 Apr 5;103(14):e37614. doi: 10.1097/MD.0000000000037614 (PMC10994480; doi:10.1097/MD.0000000000037614)
Supplement: Supplementary file 3 [file medi-103-e37614-s003.docx]

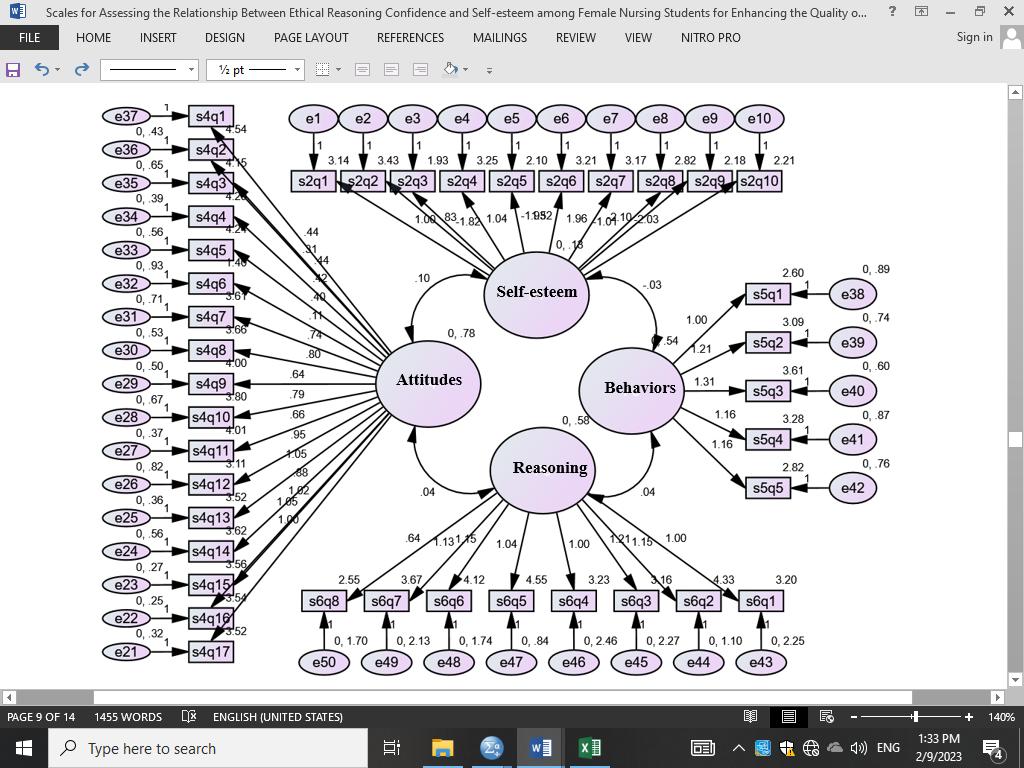


**Supplementary Figure 1: Structure Equation Modeling**

Model fit parameters CFI; IFI; RMSEA (0.646; 0.651; 0.100).

CFI = Comparative fit index; IFI = incremental fit index; and RMSEA = Root Mean Square Error of Approximation.

Model χ^2^; significance 2.610^*^(<0.001*)
